# A Rare Cause of Septicemia After Pork Meat Ingestion

**DOI:** 10.7759/cureus.62096

**Published:** 2024-06-10

**Authors:** Ricardo José Razera, José Vitor Santos-Oliveira, Matheus Boaventura-Santos, Victor Almeida-Pontes, Marcia Y Kanegae, Jose C Ardengh

**Affiliations:** 1 Digestive Endoscopy, Instituto de Infectologia Emílio Ribas, São Paulo, BRA; 2 Medicine, Instituto de Infectologia Emilio Ribas, São Paulo, BRA; 3 Medicine, Hospital das Clínicas da Faculdade de Medicina da Universidade de São Paulo, São Paulo, BRA; 4 Rheumatology, Hospital Universitário da Universidade de São Paulo, São Paulo, BRA; 5 Gastrointestinal Endoscopy, Hospital das Clínicas de Ribeirão Preto, Ribeirão Preto, BRA; 6 Image Diagnosis, Universidade Federal de Sao Paulo, São Paulo, BRA

**Keywords:** sepsis-associated disseminated intravascular coagulation (dic), toxic shock syndrome, bacterial infections, diagnosis, organ failure from sepsis, severe sepsis, streptococcus suis

## Abstract

*Streptococcus suis* infection in humans occurs due to consuming raw or undercooked pork meat and after contact with pigs. The highest prevalence occurs in Southeast Asian countries, which have the largest pork industry. We report the first case of a 50-year-old healthy male patient from a rural area of São Paulo, Brazil, with septicemia from undercooked pork meat ingestion. The patient was diagnosed at the emergency department with septicemia and multiple organ dysfunctions, including streptococcal toxic shock syndrome. Blood cultures yielded the growth of *S. suis*. The patient was treated with ceftriaxone and was maintained for two weeks, according to sensitivity tests. The outcome was favorable but developed deafness as a sequela. This report aims to give importance to recognizing this disease regarding typical signs and symptoms and occupational and epidemiological history.

## Introduction

*Streptococcus suis* (*S. suis*) is a facultative anaerobic gram-positive coccus that colonizes the upper respiratory, gastrointestinal, and urogenital tracts of pigs. In veterinary medicine, pathogenicity is seen among animals under stress or immunosuppression [1]. In the last 10 years, cases of sepsis and meningitis have been observed in humans, in which infection occurs predominantly through injured skin or the ingestion of raw or undercooked pork meat. The identification of *S. suis* occurred in more than 30 countries. Most reports concentrate on Southeast Asia, where the pork industry and meat consumption are marked. In Brazil, serotype 2, known to be the most virulent, is the most prevalent [2]. The clinical picture is characterized by septicemia with or without involvement of the cerebral meninges. *S. suis* meningitis has a lower lethality than other forms of streptococcal meningitis but has high morbidity [3]. The present report aims to describe a rare case of a previously healthy patient who presented with severe septicemic disease and auditory sequelae.

## Case presentation

**Figure 1:**
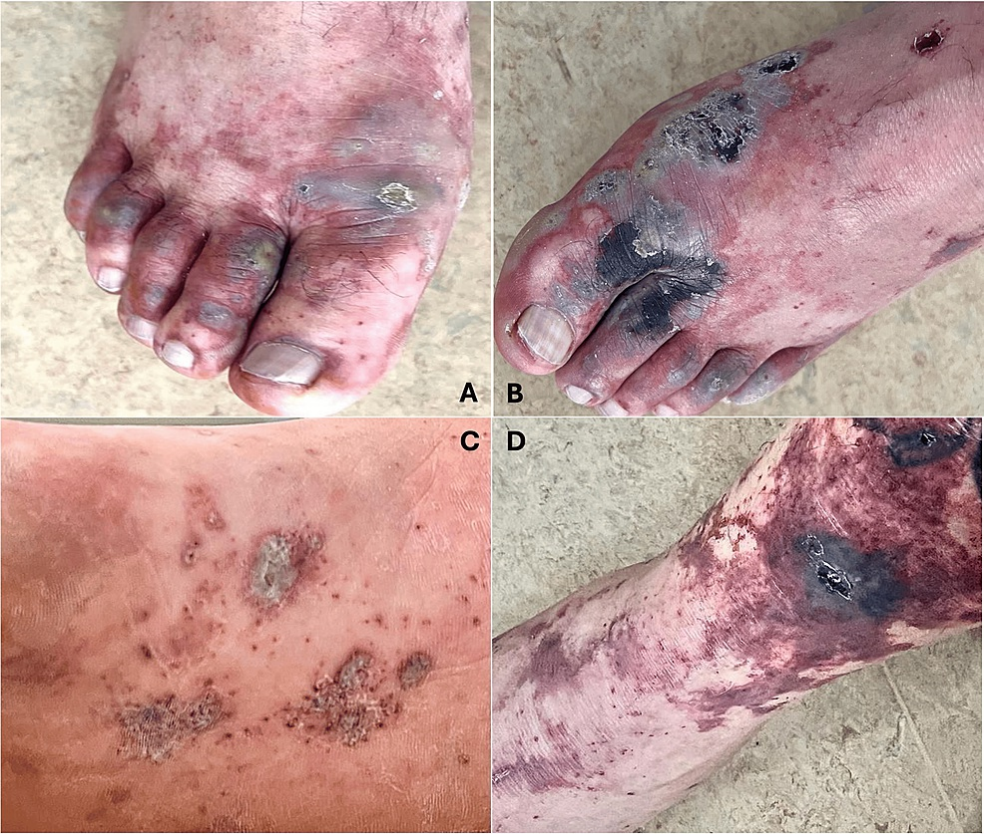
Macroscopic view of the lower limbs. Presence of hemorrhagic blisters interspersed with petechial areas that form hemorrhagic suffusions. Note: necrosis on the right fingers and foot (A) and left fingers and foot (B), sole (C), and leg (D).

**Figure 2:**
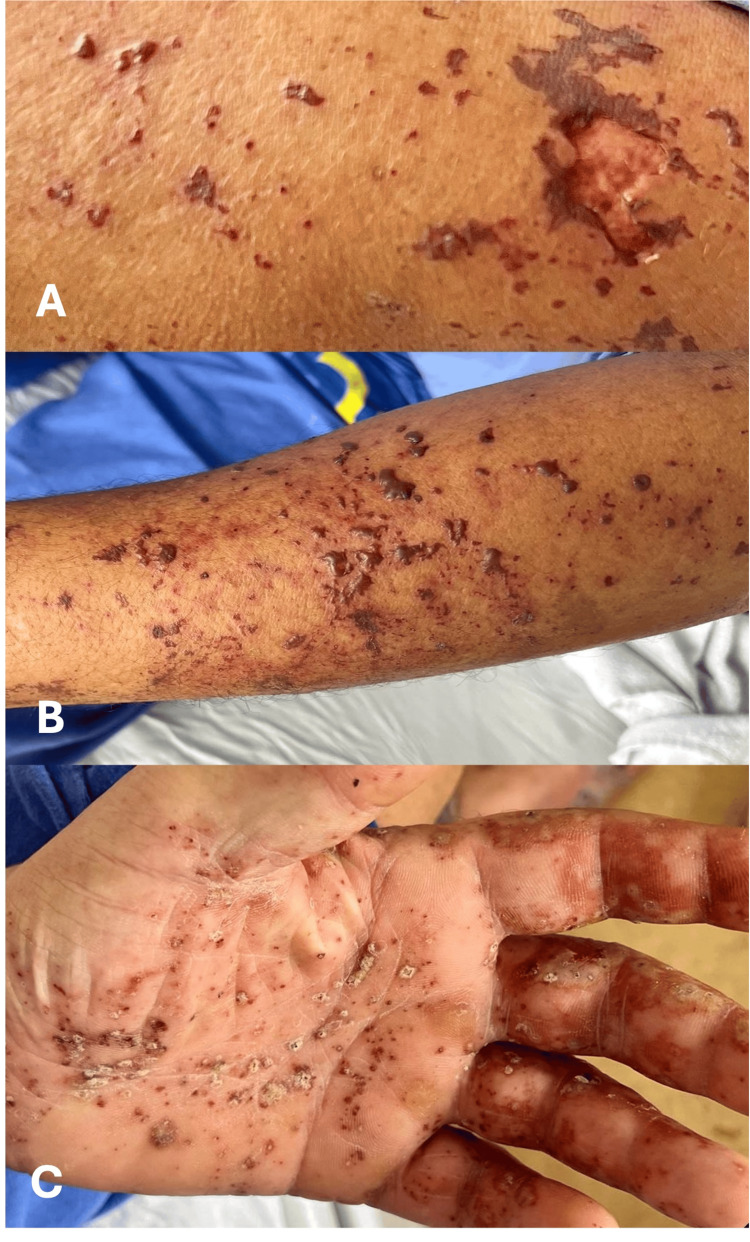
Macroscopic view of the lesions identified in the upper limbs. Note: The hemorrhagic suffusions spread over the entire surface of the arms and hands, including the palms of the hands. In (A), we observed a continuity solution with destruction of the epidermis and hemorrhagic suffusions in the dermis of the arm. (B) Hemorrhagic suffusions of varying sizes where some of them coalesce on the surface of the forearm. In the palm of the hand, we observe suffusions in different evolutionary periods.

**Figure 3:**
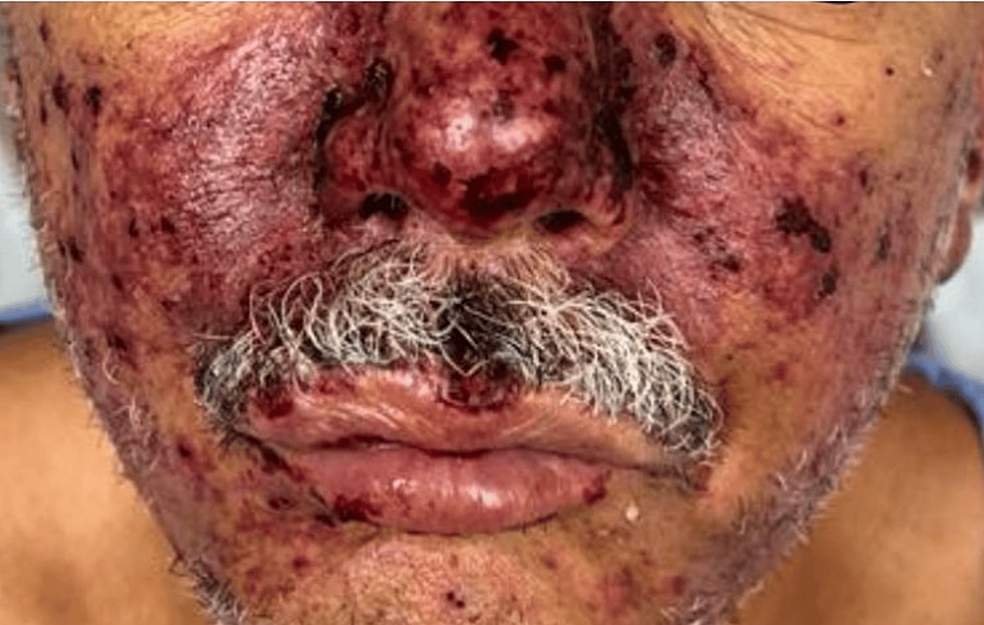
Presence of intense purpuric malar erythema, affecting the nasolabial fold.

A previously healthy 50-year-old male patient from a rural area was admitted to the emergency department complaining of diffuse abdominal pain, vomiting, diarrhea, headache, and fever. He ate raw pork meat while preparing a meal the day before. A purpuric rash in the nasal, malar region, and lower and upper limbs suddenly appeared together with skin necrosis over the extremities, accompanied by pain and slight spontaneous bleeding (Figures 1, 2, 3). The physical examination showed an ill-looking patient, with a blood pressure of 80 x 50 mmHg, pulse rate of 110 bpm, respiratory rate of 22, and room air oximetry of 93%. He was admitted to intensive care with a diagnosis of acute abdominal infection caused by severe gastroenteritis associated with septic shock. He was started on volume infusion, ceftriaxone, metronidazole, and vasoactive drugs. Laboratory workup revealed renal and hepatic dysfunctions and disseminated intravascular coagulation. Blood cultures revealed early positivity in aerobic and anaerobic bottles, identifying *S. suis* in two days.

The patient improved clinically, recovering from the renal injury and the other organic dysfunctions. The brain computed tomography was unremarkable. However, the cerebral spinal fluid (CSF) analysis, sampled after *S. suis* identification, showed high protein levels, low glucose, and cellularity, represented by lymphocytes and monocytes. CSF culture was negative. Although a favorable outcome, the patient developed a sudden sensorineural hearing loss. The dermatological lesions, attributed to toxic shock syndrome improved one week after treatment. The patient was discharged in good condition and scheduled for outpatient follow-up.

## Discussion

*S. suis* is a bacterium known for causing infections in pigs and, more rarely, humans. Classically, it causes meningitis, often followed by permanent sequelae, including deafness and ataxia. Less frequently, it may present the systemic form characterized by nausea, vomiting, high fever, subcutaneous hemorrhage, and septic shock, known as streptococcal toxic shock syndrome [4-6].

There are 35 different *S. suis *serotypes. The serotype 2 is the most prevalent and virulent in Brazil. Other specific virulence markers are identified, such as muramidase-released protein (MRP), extracellular factor (EF), hemolysin (suilysin), and capsular polysaccharide (CPS), which confers greater virulence [4,7].

In most cases worldwide, exposure to pigs or pork meat is recognized as a risk of developing the disease. Patients are young men working in pig farming or slaughterhouses, butcher shops, and veterinarians [5]. Although the most common route of exposure and infection appears to be the skin, especially during slaughter and preparation of the animal, it seems that ingestion of contaminated meat is also a route of contamination causing severe diseases [8]. The differential diagnosis needs to include epidemiological data, such as pork consumption, slaughterhouse workers, farmers, and butchers, in order to classify the individual in the risk group for *S. suis* infection in the presence of a consistent clinical picture.

*S. suis* infection became an occupational public health problem in the West, although their description in the Americas is scarce. Searching on PubMed, SciELO, and LILACS, using the keywords "sepsis," "shock,*" "Streptococcus suis*," "disseminated intravascular coagulation," "septicemia," and "meningitis," we found one article reporting two cases of* S. suis* meningitis. Thus, our report is the first description of septicemia caused by *S. suis* in Brazil [2,8,9].

*S. suis* meningitis has lower mortality than other types of bacterial meningitis; however, in the systemic infection form, the mortality is high mainly within the first 24 hours of the disease. Streptococcal toxic shock syndrome reaches 60% of mortality [8]. *S. suis* strains, in most cases, present a broad sensitivity profile, allowing treatment with penicillin, cephalosporins, carbapenems, quinolones, and tetracyclines [10,11].

Dexamethasone is not yet well-established; however, it seems to reduce sequelae and is indicated in cases with suspected infection and where the endemicity is high. Up to 53% of the affected patients experience permanent deafness after *S. suis* infection [12].

## Conclusions

*S. suis* infection is an entity in which diagnosis should be considered in endemic areas in patients with occupational and dietary exposure presenting sepsis or meningitis. The faster diagnosis and the bacteria's resistance to antimicrobial results constitute a major concern for further studies. Although the medical literature needs improvement on this entity, the available reports and reviews demonstrated better prognosis and reduced sequelae rate when the treatment was started within 24 hours of the initial symptoms. Thus, our report draws attention to the diagnosis of an emerging disease.
